# Role for CCN1 in lysophosphatidic acid response in PC‐3 human prostate cancer cells

**DOI:** 10.1002/ccs3.12019

**Published:** 2024-02-20

**Authors:** Pravita Balijepalli, Brianna K. Knode, Samuel A. Nahulu, Emily L. Abrahamson, Mary P. Nivison, Kathryn E. Meier

**Affiliations:** ^1^ Department of Pharmaceutical Sciences College of Pharmacy and Pharmaceutical Sciences Washington State University Spokane Washington USA

**Keywords:** COVID‐19, family, gender, inequality, space

## Abstract

Lysophosphatidic acid (LPA) and sphingosine 1‐phosphate (S1P) are bioactive phospholipids that act as mitogens in various cancers. Both LPA and S1P activate G‐protein coupled receptors (GPCRs). We examined the role of CCN1/CYR61, an inducible matricellular protein, in LPA‐induced signal transduction in PC‐3 human prostate cancer cells. We found that both LPA and S1P induced expression of CCN1 and CCN2 within 2–4 h. CCN1 was induced by 18:1‐LPA, but not by 18:0‐, 18:2‐, or 18:3‐LPAs. A free fatty acid receptor‐4 agonist inhibited LPA‐induced CCN1 induction. CCN1 appeared in the ECM within 2 h after LPA addition. LPA caused biphasic activation of Erk MAPK, with an initial peak at 10–20 min followed by a later phase after 6 h. LPA increased adhesion of PC‐3 cells to culture substrates (standard culture plates, fibronectin, or extracellular matrix) at 2 h, an intermediate event between early and late LPA signals. Knockdown of CCN1 suppressed LPA‐induced adhesion to ECM or fibronectin. ECM from CCN1 knockdown cells was a poor substrate for adhesion, as compared to ECM from control cells. These results suggest that CCN1 contributes to LPA responses in the tumor microenvironment. The LPA‐CCN1 axis holds promise for the development of novel therapeutic strategies in cancer.

## Graphical abstract

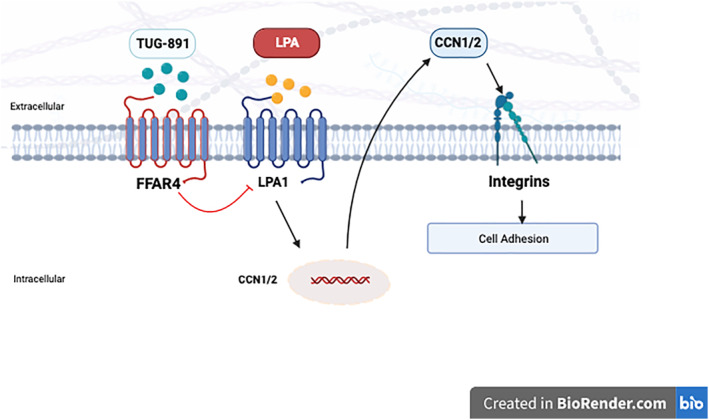

## 1 INTRODUCTION

Lysophosphatidic acid (LPA) is a bioactive phospholipid that is ubiquitously present in the human body.^1^, ^2^ The term LPA refers to the family of simple phospholipids with a single acyl or alkyl substituent; multiple species of LPA are present in biological fluids.^3^, ^4^ LPA species signal primarily through their six cognate G‐protein coupled receptors (GCPR), LPA1‐LPA6.^5^, ^6^ LPA has been implicated in various cancers for pro‐oncogenic responses that include proliferation, migration, growth, and survival.^4^, ^7^, ^8^, ^9^, ^10^, ^11^, ^12^

Sphingosine‐1‐phosphate (S1P), another bioactive phospholipid, is derived from sphingolipid metabolism.^2^ S1P acts through five S1P receptors, which are GPCRs related in sequence to LPA receptors, to exert pro‐mitogenic effects in various cancers.^13^, ^14^

Among the growth factors that play roles in cancer progression, LPA and S1P are critical contributors.^15^, ^16^, ^17^, ^18^ LPA and S1P regulate extracellular matrix (ECM), influencing its stiffness and composition, which in turn affects tumor progression and metastasis.^19^

Our research group has investigated the roles of both LPA and S1P in prostate cancer.^8^, ^20^, ^21^ Human prostate cancer cells, and particularly the PC‐3 cell line, have been used by multiple groups to study LPA responses.^9^, ^20^, ^22^, ^23^, ^24^ During our previous studies, we observed that LPA induces the production of CCN1, a matricellular protein, in PC‐3 cells.^1^ Others have shown that CCN1 is important for both proliferation and apoptosis of PC‐3 cells.^25^ For the work reported herein, we utilized the PC‐3 cell model to address the hypothesis that CCN1 plays a role in LPA‐mediated downstream responses.

Cellular communication network (CCN) proteins comprise a family of six cysteine‐rich matricellular proteins that play important roles in cellular processes such as proliferation, differentiation, adhesion, migration, and survival.^26^, ^27^ CCN1/Cyr61 (cysteine‐rich angiogenic inducer 61) and CCN2/CTGF (connective tissue growth factor) have been implicated in various stages of cancer progression, including tumor initiation, angiogenesis, invasion, and metastasis.^28^ CCN proteins modulate signaling pathways both extracellularly and intracellularly.^29^, ^30^, ^31^, ^32^

CCN‐mediated signaling mechanisms are dependent on availability of receptors as well as the individual motifs of each CCN.^33^, ^34^ All proteins in the CCN family share structural homology, and each structural motif shares similarities with those in other extracellular proteins.^35^ CCN proteins have four functional modules; Insulin‐like growth factor binding proteins (IGFBP), von Willebrand factor type C (vWC), thrombospondin (TSP), and a CT domain; the exception is CCN5, which lacks the CT domain.^36^, ^37^

CCN1 and CCN2 are encoded by early inducible genes.^1^, ^38^ Previous studies have shown that CCN1 and CCN2 play functional roles in the development of different types of cancers.^1^, ^39^, ^40^, ^42^ CCN1 can enhance adhesion of endothelial cells, fibroblasts, smooth muscle cells, monocytes, platelets, and human chondrosarcoma cells via its interaction with integrins such as integrin‐αvβ3.^43^, ^44^, ^45^, ^46^ Previous studies have shown that activation of LPA and S1P receptors can induce cellular production of CCN1 and/or CCN2 within a few hours.^1^, ^47^ This raises the possibility that CCN proteins are part of a GPCR/CCN “axis” in which CCNs perpetuate and expand upon early signals initiated by GPCR activation. In this study, we examined the role of CCN proteins in LPA response in a human prostate cancer cell line.

## 2 MATERIALS AND METHODS

### 2.1 Cell culture

PC‐3 cells were obtained from the American Type Culture Collection (Manassas, VA, USA). The cells were grown in RPMI 1640 medium supplemented with 10% (v/v) fetal bovine serum (FBS) (Hyclone/Cytiva) and 50 U/ml penicillin/50 μg/ml streptomycin. The cells were grown in an incubator at 37°C with 5% CO_2_ on standard tissue culture plastic.

### 2.2 Cell incubations for signal transduction assays

Cells were grown on standard cell culture plates in RPMI 1640 medium supplemented with 10% FBS until ∼80% confluent. Cells were serum‐starved for 24 h in serum‐free RPMI 1640 medium, then incubated with 10 μM 18:1‐LPA (Echelon Biosciences, UT), 10 μM S1P (Enzo Life Sciences, NY), and/or 1 μM TUG‐891 (Millipore Sigma, MO) for the indicated times. LPA and S1P were delivered as 1000X stock solutions prepared in 4 mg/mL fatty acid‐free BSA. Sources for other species of LPA were: 18:0‐LPA (Avanti Polar Lipids), 18:2‐LPA, and 18:3‐LPA (Echelon Biosciences, UT). Cells were rinsed twice with ice‐cold phosphate‐buffered saline (PBS), harvested by scraping into 1 mL ice‐cold PBS, collected by microcentrifugation for 10 min at 4°C, and resuspended in ice‐cold lysis buffer (20 mM HEPES [pH = 7.4], 1% Triton X‐100, 50 mM NaCl, 2 mM EGTA, 5 mM *β*‐glycerophosphate, 30 mM sodium pyrophosphate, 100 mM sodium orthovanadate, 1 mM phenylmethylsulfonyl fluoride, 10 μg/ml aprotinin, and 10 μg/ml leupeptin). Insoluble debris was removed following microcentrifugation. Protein concentrations of the supernatant “whole‐cell extracts” were determined using a bicinchoninic acid assay (Pierce).

### 2.3 Immunoblotting

Whole‐cell extracts containing equal amounts of protein (30 μg) were separated by SDS‐PAGE on 12% or 15% Laemmli gels, transferred to PVDF membranes, and incubated with primary antibody (overnight at 4°C) and then secondary antibody (1–2 h at room temperature) in Tris‐buffered saline (pH 7.8) containing BSA in (4 μg/mL) and 0.1% Tween‐20. Antibodies recognizing CCN1 and CCN2 were from Cell Signaling Technologies (#14479S; #86641S). Antibodies recognizing phosphorylated active Erk (#4370) and GAPDH were from Santa Cruz Biotechnologies. Secondary antibodies, anti‐rabbit, and anti‐mouse (IgG HRP‐linked; #7076; #7076S, respectively) were obtained from Cell Signaling Technologies. Primary and secondary antibodies were used at 1:1000 and 1:2000 dilutions, respectively. Blots were developed using enhanced chemiluminescence reagents (Pierce) and imaged using a GelDoc system (Bio‐Rad). Protein expression was quantified by densitometry using Image J software. Results were normalized to the GAPDH loading control after background subtraction.

### 2.4 Immunocytochemistry

PC‐3 cells were seeded on cover slips in 10 mm dishes in RPMI 1640 with 10% FBS, 50 U/ml penicillin/50 μg/ml streptomycin, and 2.5 μg/mL fungizone. Once 70%–80% confluence was attained, the cells were serum‐starved for 24 h. The following day, cells were incubated with and without 10 μM 18:1‐LPA for 4 h. Each plate containing the coverslip was rinsed twice with PBS, and then incubated with 4% paraformaldehyde for 15 min. Cells were then permeabilized by incubation with 1% Triton‐X in PBS for 10 min. After the permeabilization step, cells were rinsed with ice‐cold PBS, incubated for 1 h with 5%–10% FBS, and then rinsed with PBS before incubation with the primary antibody made in 1:1000 dilution in 4 mg/mL bovine serum albumin (BSA) solution overnight at 4°C. This was followed by incubation with secondary antibody (Anti‐rabbit Alexa Fluor 488; Invitrogen) for 1 h at room temperature. After the incubation, the cover slips were subjected to a series of ethanol incubations (75%, 80%, 90%, and 100%) for 1 min each. Next, the cover slips were carefully extracted from the plates and mounted on the microscope slides using mounting media containing 4′,6‐diamidino‐2‐phenylindole (DAPI; the ends were sealed using a commercial nail polish. The slides were stored at 40^o^C and were imaged using a confocal microscope (Leica CMi8 confocal microscope; WSU Health Sciences Microscopy Service Center).

### 2.5 Extracellular matrix lysates

This procedure was adapted from a published method.^48^ PC‐3 cells were grown in 100‐mm dishes in RPMI medium supplemented with 10% FBS (v/v), 50 U/ml penicillin/50 μg/ml streptomycin, and 2.5 μg/mL fungizone. After the cells reached confluence, they were serum‐starved overnight and then incubated for 2–4 h with and without 10 μM 18:1‐LPA in RPMI 1640 at 37^o^C. At the conclusion of the incubation, the medium was removed by aspiration. Cells were incubated with 2 mL of 20 mM ammonium hydroxide at room temperature for 5 min with gentle rocking. The cells were checked by microscopy for detachment and then gently aspirated using a pipette. The insoluble ECM layer was washed four times with copious deionized water to remove all the materials soluble in ammonium hydroxide. SDS‐PAGE sample buffer (200 μL) containing 100 mM dithiothreitol, heated to 95°C for 2 min, was added to each plate and the resulting samples were used for immunoblotting.

### 2.6 Adhesion assays

These assays, which were a modification of a method described previously,^49^, ^50^ were performed on 96‐well plates that were either standard cell culture plastic, or plastic coated with fibronectin (BioCoat® Fibronectin 96‐well Clear Flat Bottom TC‐treated Microplates, Corning). PC‐3 cells were grown in 100 mm cell culture plates at a seeding density of 2.2 × 10^6^ cells/well in RPMI 1640 supplemented with 10% FBS. Cells were grown to 70%–80% confluence, and then serum‐starved for 24 h. The following day, cells were detached with an addition of 0.2% trypsin EDTA, which was subsequently neutralized by addition of RPMI‐1640 supplemented with 5 mM HEPES (pH 7.5). The cells were sedimented using a microfuge for 5 min at room temperature. The cell pellet was resuspended in serum‐free RPMI supplemented with 10 mM HEPES (pH 7.4). After the cells were counted using a hemacytometer, they were resuspended in serum‐free medium with or without 10 μM 18:1‐LPA. The final cell suspension was prepared at a density of 2.5 × 10^5^ cells/ml. Next, 100 μL of cell suspension was added to each well and the plates were placed in a cell culture incubator. After each time point (0, 2, and 4 h), cells were washed once with 100 μL PBS per well and fixed using 100 μL of 0.5% crystal violet solution made in 25 mL of 100% methanol, and 75 mL of water for 10 min. The wells were then washed four times with 100 μL deionized water to remove excess dye. The plates were left to air dry overnight. The following day, 100 μL of methanol was added to each well; the plate was incubated on a rocker for 10 min at 20 oscillations/min. For standard culture plates, cells were imaged and quantified using Fuji Image J. For fibronectin‐coated plates, stained cells were analyzed for absorbance at 570 nm using a BioTek microplate reader. Background absorbance was determined using wells incubated with medium alone and was subtracted to quantify attached cells. Replicate wells (*n* = 4) were utilized for each experimental condition.

### 2.7 CCN1 knockdown

PC‐3 cells were seeded at 2 × 10^5^ cells/well in a 6‐well plate in RPMI‐1640 media with 10% FBS. CCN1‐siRNA complex, scrambled siRNA (scr siRNA) complex, and transfection medium were added to the cells (in RPMI/10% FBS), according to the manufacturer's recommendation (Santa Cruz Biotechnology) in a volume of 1 mL per well. The cells were incubated at 37°C for 5–7 h, then supplemented with additional RPMI (1 mL) with 20% FBS. After 18–24 h, the medium was changed to RPMI supplemented with 10% FBS. After 48 h, cells were serum‐starved overnight and then incubated with and without 10 μM LPA for immunoblotting or adhesion assays as described above.

### 2.8 Cell adhesion assay with CCN1 knockdown

For one series of experiments, adhesion of cells to ECM generated by PC‐3 cells was assessed. For these studies, PC‐3 cells that had been incubated with scr‐siRNA or CCN1‐siRNA, as described above, were serum‐starved for 24 h and then either (1) removed from the wells using the procedure described above for preparation of ECM lysates, to leave ECM‐coated wells, or (2) trypsinized and re‐seeded in fresh wells for 2 h before removal to leave ECM‐coated wells. In both cases, after extensive rinsing with sterile PBS, the resulting ECM‐coated plates were used for adhesion assays as described above, testing for the adhesion of serum‐starved cells pretreated with scr‐siRNA or CCN1‐siRNA. The attached cells were trypsinized and counted using a hemacytometer.

### 2.9 Statistical analysis

Quantified data were analyzed by one‐way ANOVA followed by Sidak's comparison test or *t*‐test. All analyses were performed using PRISM software (GraphPad).

## 3 RESULTS

### 3.1 Induction of CCN1 by LPAs in PC‐3 cells

Our group previously showed that 18:1‐LPA induces CCN1 within 2–4 h in PC‐3 cells,^1^ and that the LPA receptor LPA1 is predominately responsible for LPA‐induced proliferation in both PC‐3 and DU145 cells.^51^ Physiologically, LPA exists as different species with varying acyl chain lengths and degree of unsaturation. The LPA most often used in research is 18:1‐LPA, although this species is not the most efficacious for all LPA receptors.^52^ We previously demonstrated that 18:1‐LPA was optimally efficacious in activating Erk in DU145 human prostate cancer cells expressing endogenous LPA1^20^; LPAs with fatty acid substituents containing <18 or >18 carbons were less active. For the current study, we characterized the induction of CCN1 by various LPA species in PC‐3 cells, focusing our attention on the position of the double bond in 18‐carbon LPAs.

**FIGURE 1:**
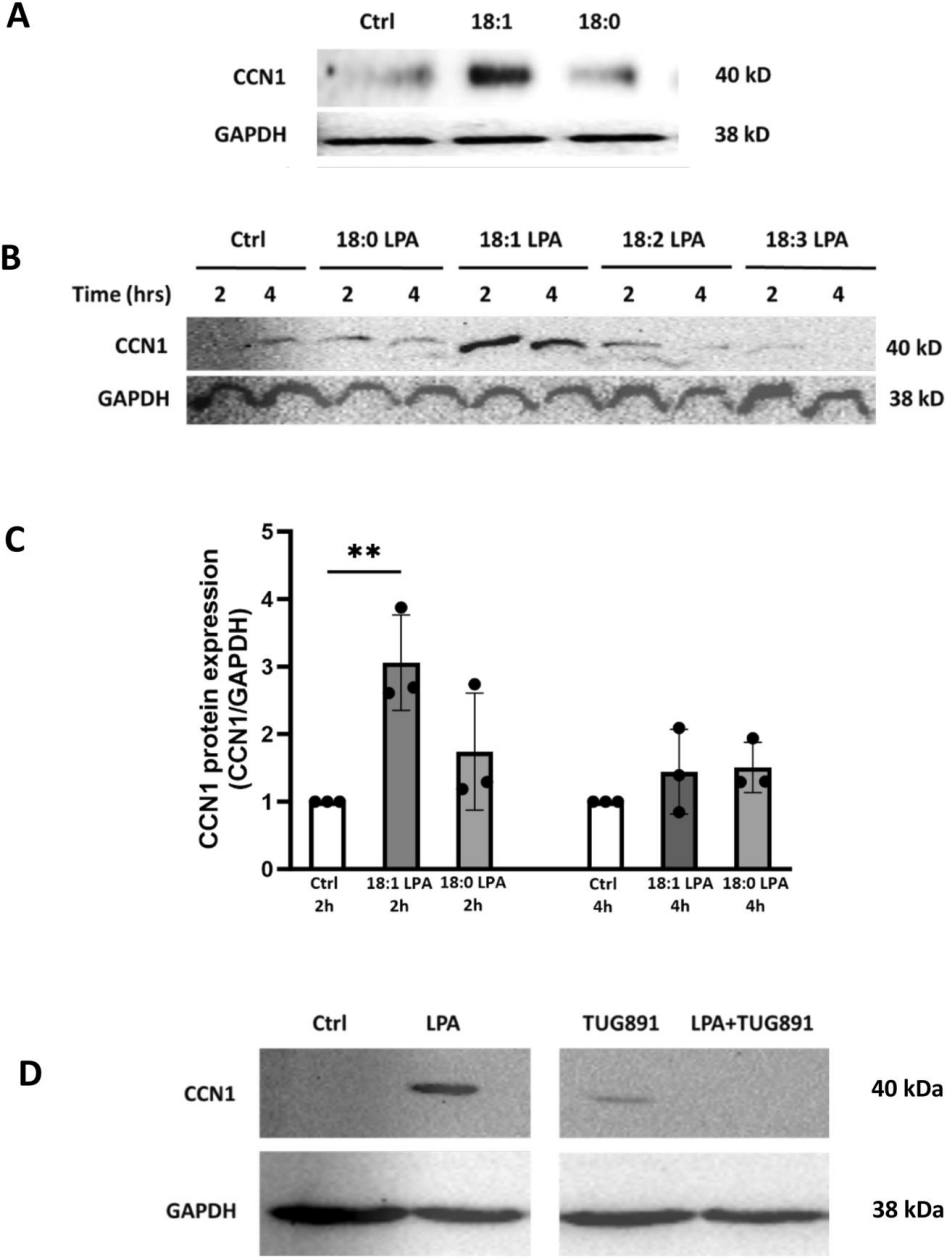
Effects of LPA on CCN1 protein levels. Panel A and B: PC‐3 cells were serum‐starved for 24 h, then incubated with or without (“Ctrl”) the indicated species of LPA (10 μM) for 2 or 4 h. Cells were harvested, and whole‐cell lysates were subjected to immunoblotting for CCN1 and GAPDH (loading control; run on a separate gel). Panel C: Immunoblot results for the effects of 18:0‐ and 18:1‐LPA on CCN1 expression, normalized to GAPDH, were quantified from three independent experiments (*n* = 3). **p < 0.01. Panel D: Serum‐starved PC‐3 cells were incubated with 10 μM 18:1‐LPA ±1 μM TUG‐891 (FFAR agonist) for 3.5 h. Whole‐cell lysates were subjected to immunoblotting for CCN1 and GAPDH (loading control; run on a separate gel). All samples for each blot were run on the same gel and were transferred, blotted, and imaged together; irrelevant lanes were removed from the center of the image. The results are representative of three independent experiments.

As shown in Figure 1A, 18:1‐LPA induced CCN1 protein expression after 2 h when added to serum‐starved PC‐3 cells, confirming our previous findings for PC‐3 and DU145 cells^1^; 18:0‐LPA had a negligible effect. In Figure 1B, additional 18‐carbon species of LPA with unsaturation at various positions were tested at both 2 and 4 h. These results demonstrate that 18:1‐LPA is more efficacious in inducing CCN1 than 18:0‐, 18:2‐, or 18:3‐LPA. In Figure 1C, quantified results from multiple experiments show that the response to 18:1‐LPA is maximal at 2 h, and that 18:0 LPA does not induce CCN1 to a statistically significant extent at either 2 or 4 h. Thus, the single double bond in 18:1‐LPA is critical for its activity and 18:1‐LPA is the appropriate ligand for further examination of CCN1 induction in PC‐3 cells. Hereafter, “LPA” will refer to 18:1‐LPA.

Previous work in our group demonstrated that agonists for another GPCR, FFAR4, inhibit downstream responses to LPA in human prostate cancer cells as well as in breast and ovarian cancer cells.^1^, ^51^, ^53^ In these studies, we found that eicosopentanoic acid (EPA), a dietary FFAR agonist, inhibited CCN1 induction in DU145 human prostate cancer cells.^1^ In the current study, the effects of a pharmacologic FFAR4 agonist, TUG‐891, were tested in the PC‐3 cell line (Figure 1D). TUG‐891 alone caused a slight induction of CCN1, consistent with the fact that FFAR4 activates downstream signaling pathways.^54^, ^55^ However, TUG‐891 blocked the ability of LPA to induce CCN1 in PC‐3 cells; the combination of the two agonists resulted in no CCN1 induction. This result is consistent with our previous findings that the concomitant administration of LPA and FFAR4 agonists blocks LPA1‐mediated signaling.^21^

### 3.2 Time course of the effects of LPA and S1P on CCN induction

**FIGURE 2:**
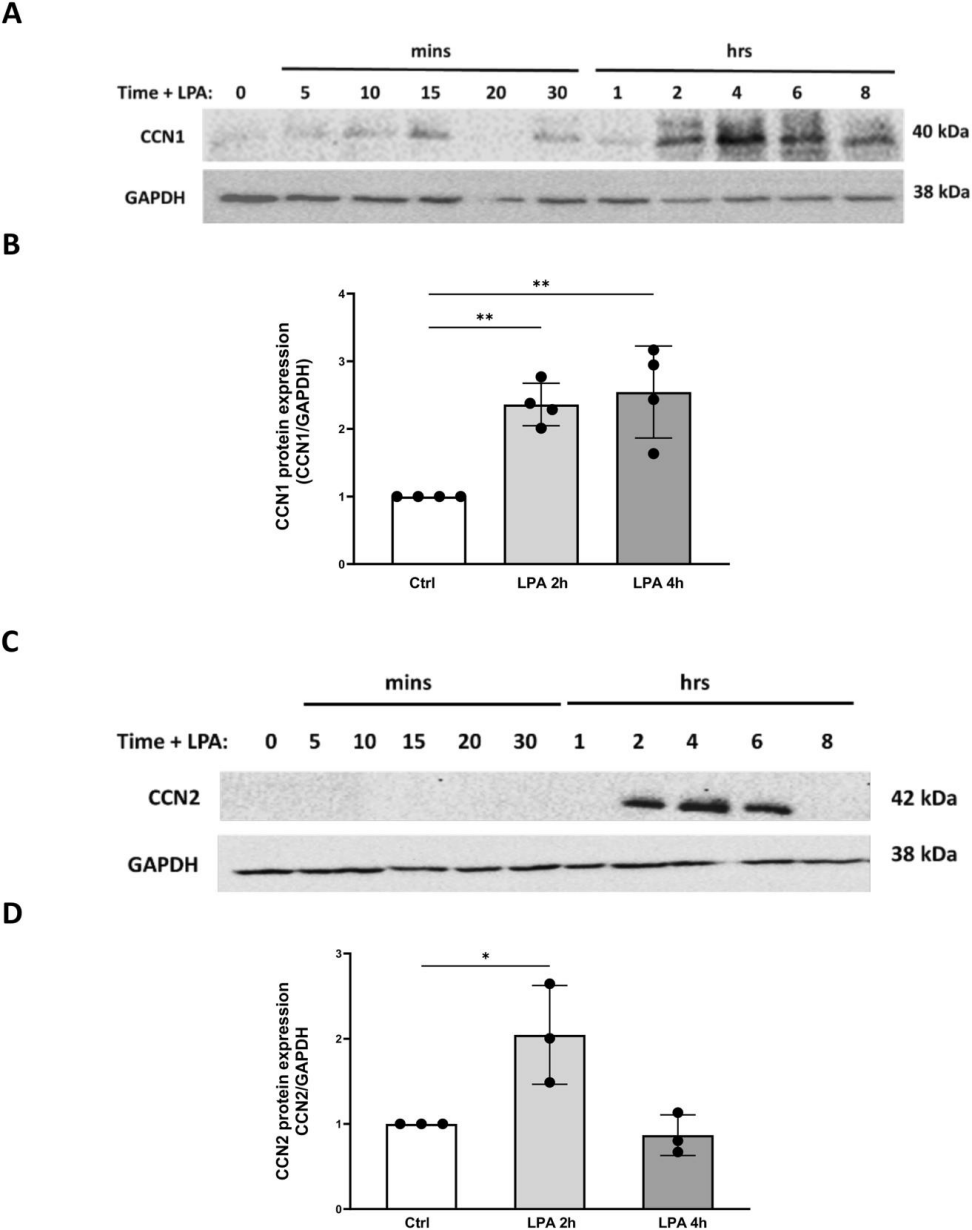
Time course of the effects of LPA on CCN1 and CCN2 in PC‐3 cells. Panels A and C: PC‐3 cells were seeded in 6‐well plates, serum‐starved overnight, and then incubated with 10 μM 18:1‐LPA for 0–8 h. Whole cell lysates were collected and subjected to immunoblot analysis. Panels B and D: CCN protein expression for each immunoblotted sample was normalized to GAPDH, using Image J, after background subtraction. Each data point represents mean ± SD of values from three separate experiments. * = *p* < 0.05; ** = *p* < 0.01.

As mentioned earlier, both CCN1 and CCN2 are inducible proteins. Effects of LPA on CCN2 expression were not examined in our previous studies. We therefore examined the time course of the effects of LPA on protein levels for both CCN1 and CCN2 using immunoblotting of whole‐cell extracts (Figure 2A–D). LPA induced CCN1 at 2–6 h (Figure 2A), consistent with previous results reported for both PC‐3 and DU145 cells.^1^ A statistically significant increase in CCN1 was observed after 2 and 4 h in these experiments (Figure 2B). LPA also induced production of CCN2 (Figure 2C). The increase in CCN2 was statistically significant 2 h after LPA addition (Figure 2D).

**FIGURE 3:**
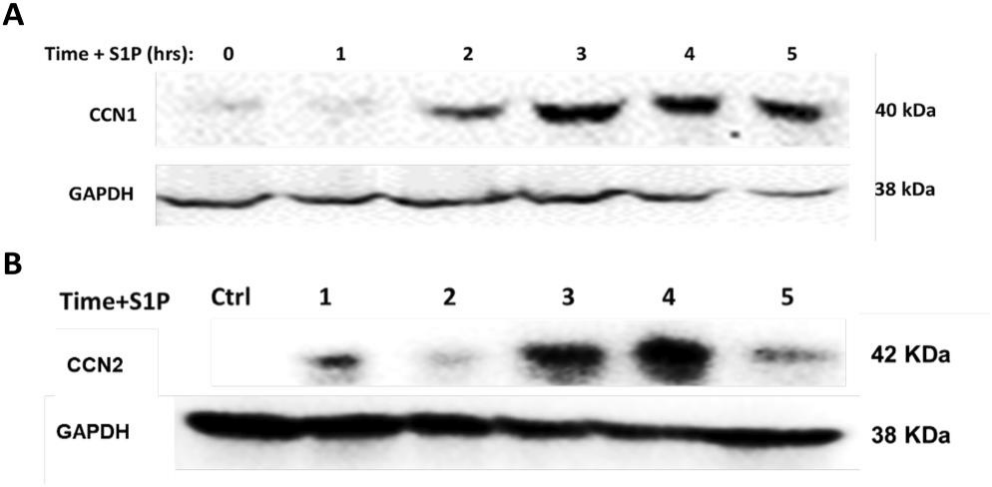
Time course of the effects of S1P on CCN1 and CCN2 in PC‐3 cells. PC‐3 cells were seeded in 6‐well plates, serum‐starved, and then incubated with 10 μM S1P for 0–5 h. Whole‐cell lysates were collected and subjected to immunoblot analysis for CCN1 (Panel A), CCN2 (Panel B), and GAPDH (loading control; run on a separate gel for both Panels A and B). The results shown are representative of three independent experiments.

Sphingosine 1‐phosphate (S1P) can induce CCN proteins in other cell types.^5^, ^56^, ^57^, ^58^ The effects of S1P were therefore tested in PC‐3 cells. As shown in Figure 3, S1P induced both CCN1 and CCN2 after 2–4 h, similar to the results observed for LPA (Figure 2). Protein levels for CCN1 were maintained longer than the levels for CCN2, also consistent with the time course for LPA.

### 3.3 Localization of LPA‐induced CCN1

**FIGURE 4:**
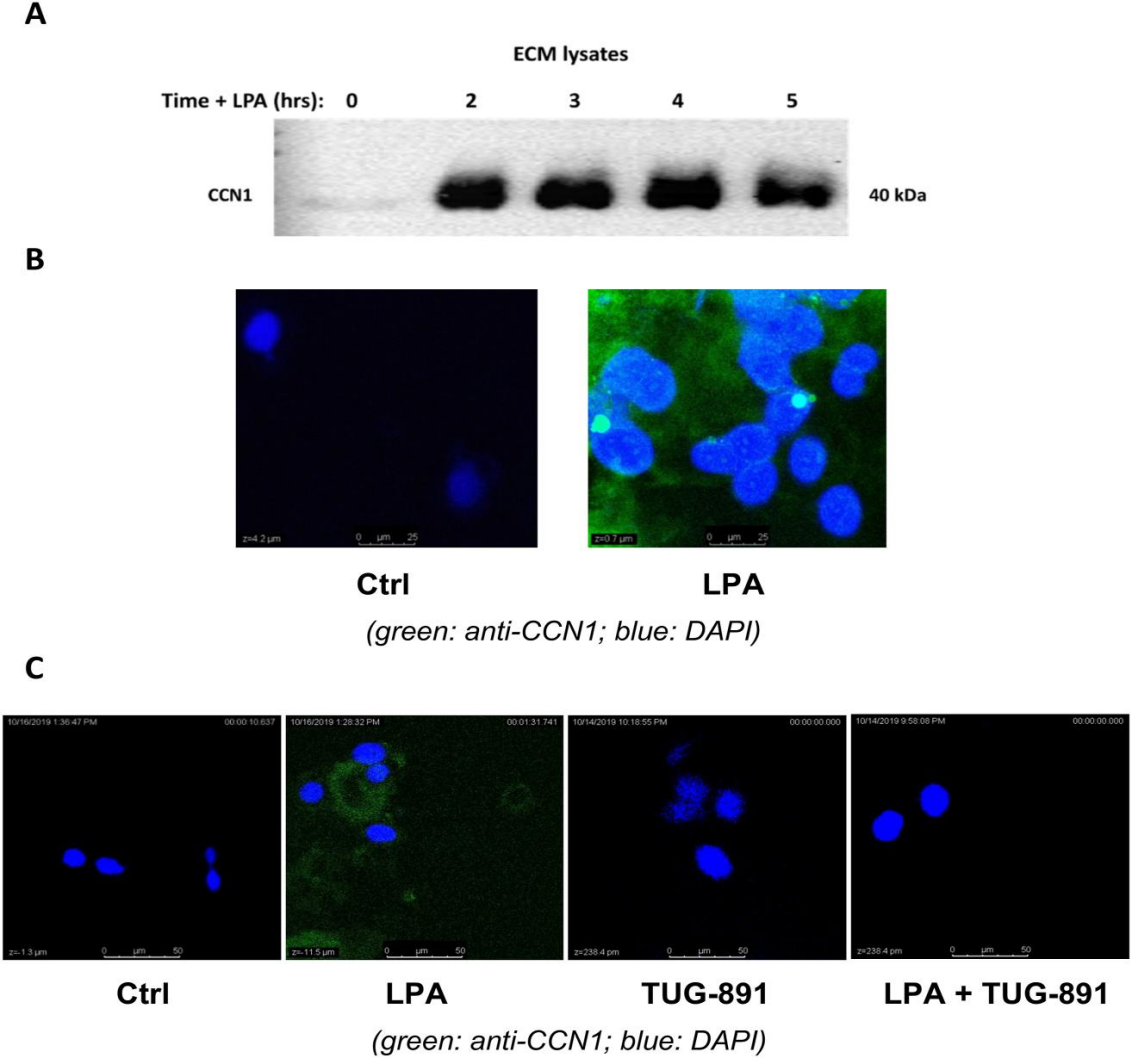
Localization of LPA‐induced CCN1. Panel A: Serum‐starved PC‐3 cells, seeded in equal numbers, were incubated with 10 μM LPA for 4 h. Cells were then treated with 20 μM ammonia to dissociate cells and retain ECM. The resulting ECM lysates were harvested and subjected to immunoblotting. The results are representative of three independent experiments. Panel B: PC‐3 cells grown on cover slips were serum‐starved for 24 h, and then treated ±10 μM LPA for 4 h. Cells were then fixed, incubated with anti‐CCN1 and DAPI, processed, and imaged using confocal immunofluorescence microscopy. Panel C: The immunolocalization experiment, as described for Panel B, was carried out in the absence and presence of the FFAR4 agonist, TUG‐891 (1 μM).

CCN proteins are secreted matricellular proteins composed of modular motifs that allow them to interact with integrins and ECM proteins. We therefore investigated the localization of LPA‐induced CCN1 protein. In the first set of experiments (Figure 4A), serum‐starved PC‐3 cells were treated with 10 μM 18:1‐LPA for 0–5 h. Extracellular matrix extracts were prepared and immunoblotted for CCN1. The results show that CCN1 was present at greatly increased levels (compared to control) in the extracellular matrix between 2 and 5 h, indicating that CCN1 was exported from the cells promptly following its LPA‐induced expression. In the next experiments (Figure 4B), confocal immunofluorescence microscopy was used to localize CCN1 in serum‐starved PC‐3 cells treated with and without LPA for 4 h. These results indicate that the LPA‐induced CCN1 was diffusely localized throughout the extracellular space, consistent with the immunoblotting results. In Figure 4C, the effects of the FFAR4 agonist, TUG891, were tested. In this experiment, LPA induced CCN1 in extracellular matrix in the absence of TUG891, but not in its presence.

### 3.4 Time course of the effects of LPA on Erk MAPK activation

**FIGURE 5:**
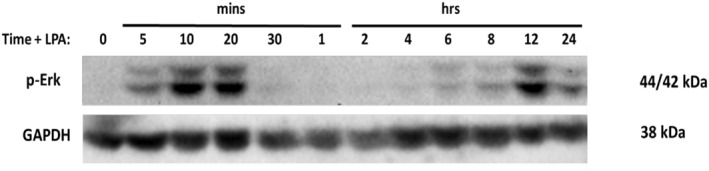
Time course of the effects of LPA on Erk activation. PC‐3 cells were serum‐starved for 24 h and then incubated with 10 μM 18:1‐LPA for 0–12 h. Whole‐cell lysates were separated on 12% SDS gels and immunoblotted for activated phospho‐Erk and GAPDH (loading control). A representative blot from three separate experiments is shown.

Since at least 1 hour is required for the LPA to increase cellular CCN1/2 levels, we investigated which other LPA‐induced signal transduction events might occur over a prolonged time course. It is well established that LPA activates the Erk MAPK pathway in prostate cancer cells.^8^, ^24^, ^59^ This response is observed within a few minutes after LPA treatment. However, it has long been known that mitogens can elicit a biphasic activation of Erk.^60^ We therefore examined the extended (24‐h) time course of Erk activation in PC‐3 cells. As shown in Figure 5, there was biphasic activation of Erk after the addition of 10 μM 18:1‐LPA. The initial response was maximal by 10 min and subsided by 30 min. The secondary phase of Erk activation occurred between 6 and 24 h and was maximal at 12 h. These results illustrate that LPA‐induced Erk activation, which is important for cell proliferation, occurs both acutely and then again ∼12 h after the initial stimulus.

### 3.5 Effect of LPA on cell‐substrate adhesion

**FIGURE 6:**
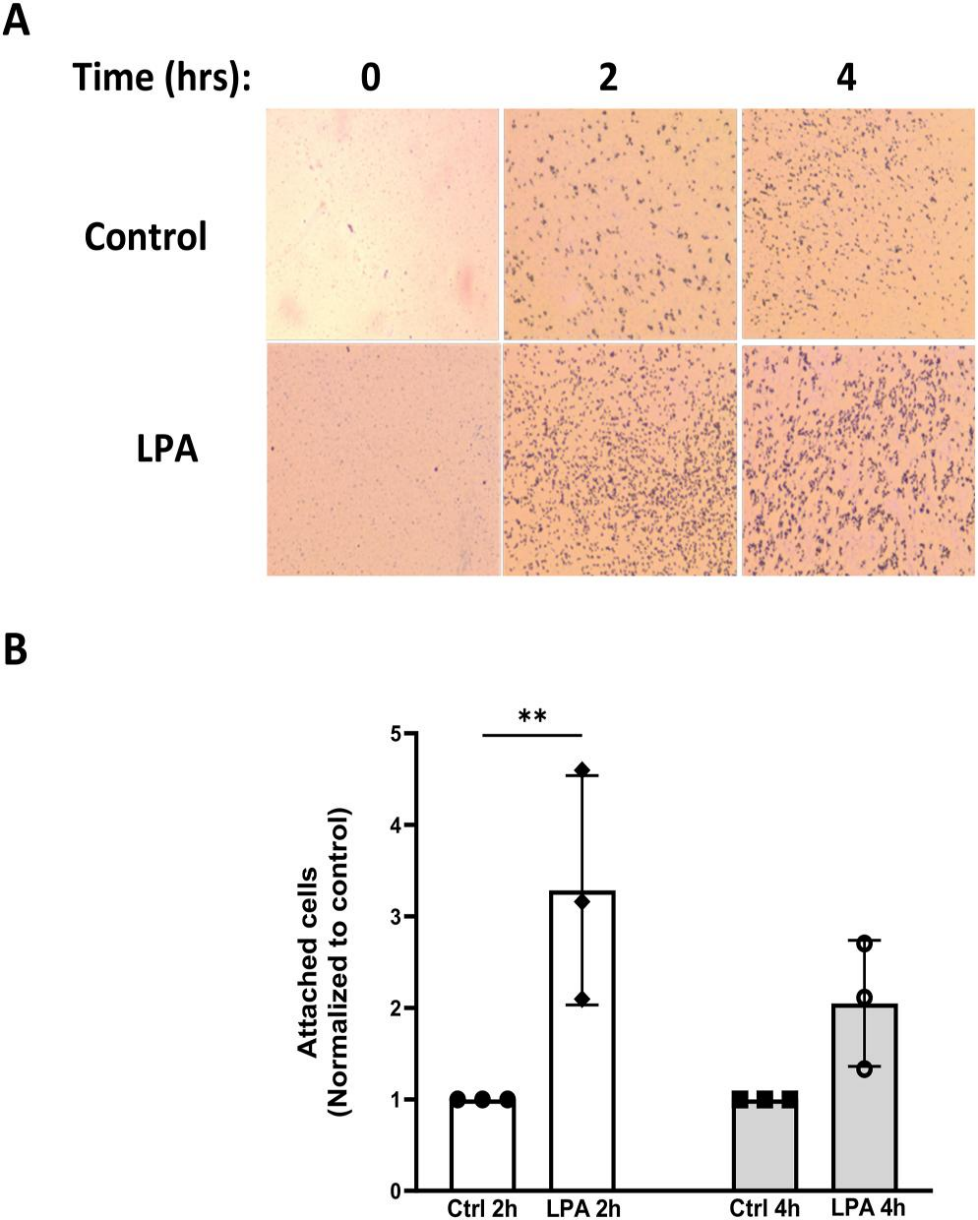
Effects of LPA on PC‐3 cell adhesion to standard cell culture plates. Panel A: PC‐3 cells were serum‐starved for 24 h, detached using trypsin, and then resuspended in serum‐free medium. Cells were seeded on standard cell culture plates and incubated with and without 10 μM 18:1‐LPA for 0–4 h. The wells were washed, stained with crystal violet, fixed, and imaged using light microscopy. Panel B: Attached cells were stained as for Panel A; attached cells were quantified using Fuji Image Lab. All values are normalized to the untreated (‐LPA) control for the corresponding time point and represent mean ± SD from three separate experiments.**p < 0.01.

Our group previously demonstrated that 18:1‐LPA increases migration of PC‐3 cells, as assessed after 6 h.^21^ However, cell‐substrate adhesion was not examined in our earlier study. Since CCNs are known to be involved in cell adhesion, we optimized an adhesion assay and used it to examine the effects of LPA. We seeded serum‐starved PC‐3 cells on uncoated cell culture plates in the absence and presence of 18:1‐LPA. At the conclusion of the incubation, cells were washed and stained for the quantification of absorbance. The results of time course experiments, as shown in Figure 6A and quantified in Figure 6B, indicated that LPA increased adhesion as assessed at 2 hours. By 4 hours, untreated and LPA‐treated cells attached to statistically similar extents. Thus, LPA transiently enhances the initial phase of cell adhesion.

### 3.6 Effects of CCN1 knockdown on cell adhesion to fibronectin

**FIGURE 7:**
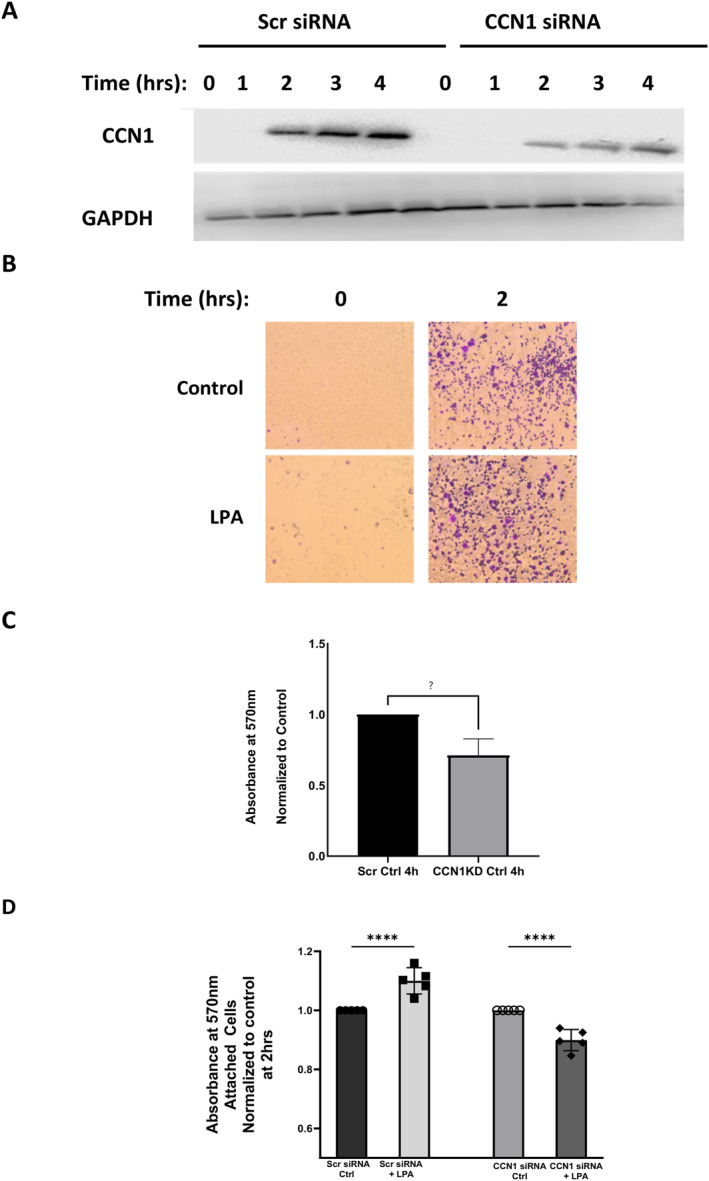
Effects of CCN1 knockdown on LPA‐induced cell adhesion to fibronectin‐coated plates. Panel A: PC‐3 cells were incubated with scrambled (“scr”) siRNA or CCN1‐siRNA, maintained for 48 h in serum‐free medium, and then incubated on fibronectin‐coated plates ± 10 μM LPA for 2–4 h. Whole‐cell extracts were immunoblotted for CCN1 and for GAPDH (loading control) on separate gels. Panel B: Cells (without siRNA treatment) were subjected to an adhesion assay in serum‐free medium ± LPA on fibronectin‐coated plates for 2 h. Attached cells were stained with crystal violet and imaged using light microscopy. Panel C: Cells incubated with scr‐siRNA or CCN1‐siRNA were subjected to the adhesion assay for 4 h using fibronectin‐coated plates, as described for Panel B. Absorbance was measured at 570 nm, comparing basal adhesion between cells incubated with scr‐siRNA and CCN1‐siRNA. All values are normalized to the control (scr‐siRNA). Panel D: Adhesion assays were carried out as described for Panel C, except that cells treated with scr‐siRNA and CCN1 RNA were incubated for 2 hours with and without 10 μM LPA. For both Panels C and D, the values represent mean ± SD from four separate experiments, each conducted with duplicate wells of cells.****p < 0.001.

We first optimized conditions for siRNA‐mediated knockdown of CCN1 in PC‐3 cells. A scrambled siRNA was used as a negative control. As shown in Figure 7A, the conditions used for the CCN1‐siRNA were successful in suppressing CCN1 expression.

For the next series of adhesion assays, we used fibronectin‐coated plates to better mimic the microenvironment in which CCNs engage with ECM proteins. The imaged results show that LPA enhanced adhesion of untreated PC‐3 cells to the fibronectin‐coated substrate after 2 h (Figure 7B).

Next, we performed cell adhesion assays on fibronectin‐coated plates using PC‐3 cells with and without knockdown of CCN1. A high‐throughput method (microplate absorbance reading) was used to quantify the results. Our first observation was that basal cell adhesion, in the absence of LPA, was significantly reduced in cells subjected to CCN1 knockdown for 4 h (Figure 7C). This result indicated that CCN1 plays a role in adhesion of PC‐3 cells to fibronectin. Since the majority of the cells were still able to attach after CCN1 knockdown, we were able to conduct further experiments to analyze the effects of CCN1 loss on LPA response.

In Figure 7D, the effects of LPA on cell adhesion to fibronectin‐coated plates were assessed after 2 h in PC‐3 cells with and without knockdown of CCN1. This experiment again utilized the high‐throughput method to measure stained adherent cells. Since CCN1 knockdown alone reduced basal adhesion (Figure 7C), the effects of LPA were normalized to the untreated controls for each condition. The results in Figure 7D show that LPA increased the adhesion of cells expressing CCN1, but did not stimulate adhesion of cells in which CCN1 expression was suppressed. Instead, LPA decreased adhesion of CCN1‐deficient cells. Taken together, the results presented in Figure 7 indicate that CCN1 is involved in basal adhesion, and is required for LPA‐enhanced adhesion.

### 3.7 Effects of CCN1 knockdown on cell adhesion to ECM produced by PC‐3 cells

**FIGURE 8:**
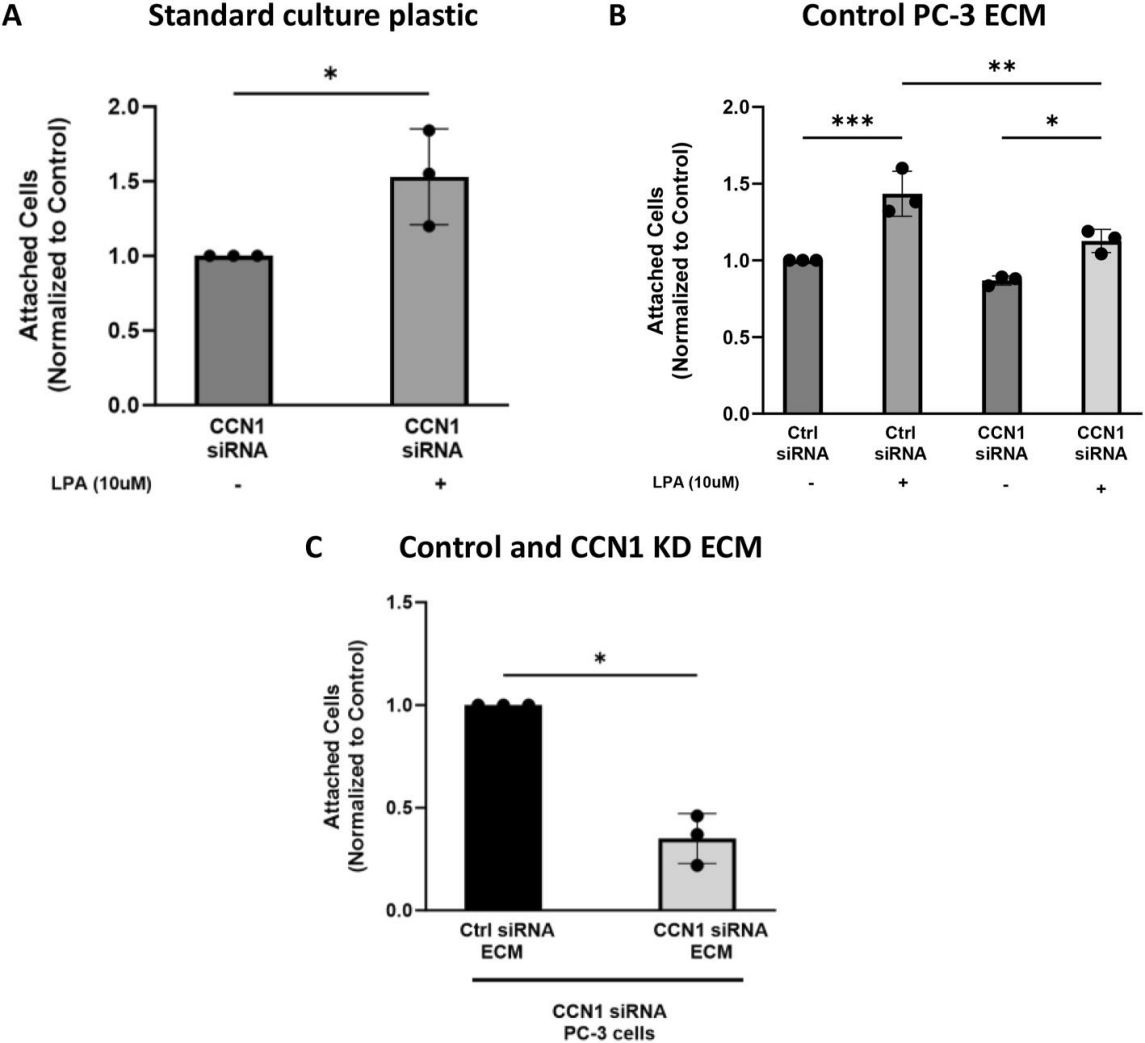
Adhesion of cells with and without CCN1 knockdown to ECM produced by PC‐3 cells. Panel A: PC‐3 cells that had been incubated with CCN1‐siRNA were used for an adhesion assay on standard culture plastic, using an incubation of 2 h ±10 μM LPA. Panel B: ECM was generated by untreated PC‐3 cells (without siRNAs) grown in wells. Cells incubated with scr‐siRNA or CCN1‐siRNA were added to the ECM‐coated wells, and incubated ± LPA for 2 h for an adhesion assay. Panel C: ECM was generated in wells in which PC‐3 cells treated with scr‐siRNA or CCN1‐siRNA had been grown. Cells incubated with CCN1‐siRNA were seeded on the ECM‐coated wells and incubated (without LPA) for 2 h for an adhesion assay. For all panels, attached cells were harvested by trypsinization and counted using a hemacytometer. Results were normalized to the respective control. In all cases, each bar represents mean ± SD of values from three separate experiments.***p < 0.001**p < 0.01, *p < 0.05.

In the next set of experiments, we tested whether CCN1 is required for the production of ECM that supports cell adhesion. First, we established that incubation with LPA for 2 h can induce adhesion of PC‐3 cells to standard tissue culture plastic after CCN1 knokdown (Figure 8A). This was similar to the situation for PC‐3 cells without CCN1 knockdown, as previously shown in Figure 6, except that the magnitude of LPA response was lower after CCN1‐siRNA.

Next, we tested the adhesion of cells, treated with and without CCN1‐siRNA, to an ECM substrate generated by untreated PC‐3 cells (Figure 8B). This substrate was anticipated to contain low levels of CCN1 (see Figure 4A). LPA enhanced the adhesion of control cells to the ECM, but there was no statistically significant response to LPA in CCN1 knockdown cells.

In the last set of experiments, ECM was generated by control and CCN1 knockdown cells over a 2‐h incubation. Basal adhesion of CCN1 knockdown cells to the ECM‐coated wells was assessed (Figure 8C). The results indicate that the ECM produced by CCN1 knockdown cells was a poor substrate for adhesion of CCN1‐deficient cells, as compared to the ECM produced by control cells. Taken together, the results presented in Figure 8 demonstrate that CCN1 expression is important for the assembly of an ECM that supports adhesion.

## 4 DISCUSSION

CCN matricellular proteins have emerged as important regulators of a wide range of cellular processes, including cell proliferation, migration, and differentiation.^61^, ^62^ In this study, we aimed to elucidate the roles of CCN proteins in LPA signaling. CCN1 and CCN2 have been implicated in responses in breast and prostate cancer cells that include proliferation, migration, survival, and tumorigenesis.^63^, ^64^ Previous studies have reported that GPCR ligands, including LPA and S1P, can induce expression of extracellular proteins including CCN1 and CCN2.^65^, ^66^ We established in the current study that both LPA and S1P induce CCN1 and CCN2 protein expression in PC‐3 human prostate cancer cells within 2 h. This suggests that LPA and S1P induce the transcription of the primary response genes encoding CCN1 and CCN2 in PC‐3 cells.

The transcriptional events responsible for CCN1 and CCN2 induction have been characterized by others. Yes‐associated protein (YAP), a transcription co‐activator that is activated by dephosphorylation, enhances transcription of both CCN1 and CCN2^35^ LPA can activate YAP in a variety of cell types.^35^, ^68^, ^69^ S1P also activates YAP.^44^, ^70^, ^71^ Our group has previously characterized the expression of multiple LPA and S1P receptors in human prostate cancer cells,^8^ and showed that LPA1 is responsible for LPA‐induced proliferation in PC‐3 cells.^21^ LPA1 can activate YAP in other cell types.^72^ PC‐3 cells express S1P1, S1P2, and S1P3,^8^ all of which can activate YAP.^71^, ^73^, ^74^ Thus, it is likely that both LPA and S1P induce CCN proteins via a YAP‐dependent pathway in PC‐3 cells.

We report for the first time, that 18:1‐LPA and 18:0 LPA have differential effects on CCN1 induction in prostate cancer cells, with 18:1‐LPA being more efficacious. While 18:1‐LPA remains the most studied LPA species, multiple LPA species are present in biological fluids^3^, ^4^ and can elicit cellular responses.^20^ The LPA receptor LPA1 is responsible for LPA‐induced proliferation and migration in PC‐3 and DU145 prostate cancer cells.^21^ We previously demonstrated that 18:1‐LPA is the most efficacious species for Erk activation in DU145 cells.^20^ The efficacy of 18:1‐LPA in inducing CCN1 expression in PC‐3 cells is consistent with its ability to activate LPA1.

CCN proteins are emerging therapeutic targets in various pathological conditions, including cancer.^34^ Several strategies can be considered as ways to interfere with the action or expression of CCNs. Antibodies targeting CCNs have shown promise in preclinical studies using a pancreatic cancer model.^75^ Our group previously reported that TUG‐891, a synthetic FFAR4 agonist, can inhibit LPA‐induced responses in prostate and breast cancer cells via a pathway mediated by FFAR4.^21^ In the current study, we observed that TUG‐891 inhibited LPA induction of CCN1 in PC‐3 prostate cancer cells. This result confirms our observations that were made previously in other cell lines and suggests that FFAR agonists deserve further investigation for therapeutic activity against cancer.^76^

Our results support the role of CCN1 as a matricellular protein that is an important downstream component of LPA‐initiated signaling pathways. The signaling roles of CCN1 have been reviewed by others.^45^, ^77^ CCN proteins share a modular structure, containing highly conserved domains: insulin‐like growth factor‐binding protein (IGFBP), von Willebrand factor type C repeats (vWC), thrombospondin type I repeats (TSR), and a carboxyl‐terminal (CT) containing a cysteine knot motif. Each domain is encoded by a different exon and is responsible for a different cellular outcome, as each domain interacts with a range of binding partners.^36^, ^78^ Previous studies have reported that CCN1 binds to ECM proteins such as fibronectin and to cell surface receptors such as integrins, supporting its role in cell adhesion in adherent cells.^61^, ^62^, ^79^, ^80^ Although CCNs are considered matricellular proteins, CCN1 has been detected in cell nuclei as well as in extracellular matrix.^81^ In our localization experiments, LPA induced CCN1 was found in the extracellular matrix of PC‐3 cells after 2–4 h. However, these results do not exclude intracellular roles for CCN1 or CCN2 in these cells.

In prostate cancer cells, LPA has been shown to promote rapid activation of Erk, a mitogen‐activated protein kinase that plays a role in cell proliferation, migration, and survival.^82^ Our current results show that a second “late‐phase” Erk activation occurs 4–12 h after LPA addition. This delayed Erk activation, seen after CCN induction, serves as another example of longer‐term responses to LPA. CCN proteins have been shown to play a role in Erk activation. CCN1 can promote cell adhesion through interactions with integrins (e.g., αvβ3 and α6β1),^65^ which are cell surface receptors involved in focal adhesion formation and cell‐matrix interactions. Engagement of integrins has been shown to result in Erk activation.^83^ Thus, it is conceivable that CCN1 and/or CCN2 can contribute to late‐phase ERK activation through their interactions with integrins.

We hypothesized that CCN1 is a key factor in LPA‐induced responses. Although CCN1 and CCN2 are associated with ECM components, they are also involved in signal transduction. For example, CCN1 has been reported to play a role in endothelial cell adhesion through binding to αvβ3 integrin.^65^ Our observations that LPA induced CCN1 expression between 2 and 6 h, and that CCN1 was detected in ECM, led us to test whether CCN1 contributes to cell‐substrate adhesion in prostate cancer cells.

The current study identified an important role for CCN1 in LPA‐induced cell adhesion. Previous work from our group demonstrated that the effects of LPA on prostate cancer cell adhesion are complex. LPA induces rapid (within 15 min) phosphorylation of focal adhesion kinase (FAK) in PC‐3 and DU145 cells.^8^ In PC‐3 cells, where LPA protects FAK from ionomycin‐induced proteolysis, LPA causes cell rounding within 7 min in the absence or presence of serum^84^; this response is an early event in a series of adhesion‐related responses occurring after LPA receptor activation. In the current study, we examined later events downstream of LPA, and found that LPA increased cell adhesion as assessed after 2 h, using standard culture plastic, fibronectin‐coated plastic, or ECM‐coated plastic.

We observed that LPA‐enhanced adhesion to fibronectin or ECM was reduced or absent after CCN1 knockdown (Figures 7 and 8). In addition, ECM produced by CCN1 knockdown cells was deficient in terms of supporting adhesion. Taken together, the adhesion assay results demonstrate that LPA‐mediated CCN1 upregulation plays a role in cell‐substrate adhesion.

There are several aspects of CCN function that remain to be explored. The role of CCN2, which is also induced by LPA, was not tested in the current study; we acknowledge that CCN1 and CCN2 share some functionalities and that both proteins are likely important to LPA response. In addition, CCN1 and CCN2 are proteolyzed to generate fragments with bioactivity^85^; this aspect was not examined in our study. Potential interactions between CCN1 and CCN2, and between their proteolysis products, add complexity when both proteins are induced by the same stimulus. Finally, it is important to note that CCN1 can play roles within the cells where it is produced, in addition to its extracellular role^79^; the intracellular roles of CCN proteins are still being elucidated and were not examined here. Further investigations are warranted to elucidate the functional consequences of CCN1 and CCN2 induction in PC‐3 and in other prostate cancer cell lines.

In summary, our study provides key evidence that CCN1 plays an important role in the LPA downstream signaling in prostate cancer cells. CCN2, which is co‐induced by LPA, may play a related role. This work provides an example of how CCNs can participate sequentially in an “LPA/CCN axis” of signal transduction. These results further illustrate the role of CCNs as emerging therapeutic targets in cancers. Our cumulative studies show that LPARs and CCNs can both be targeted using FFAR agonists or LPA antagonists; these are pharmacologic agents that are in the drug development pipeline. Our work emphasizes the need for further investigation of the potential roles of additional receptors and signaling proteins in the “GPCR‐CCN” axis.

## AUTHOR CONTRIBUTIONS

**Pravita Balijepalli**: Conceptualization; data curation; formal analysis; investigation; methodology; validation; visualization; writing—original draft preparation; writing—reviewing & editing. **Brianna K. Knode**: Investigation; writing—reviewing & editing. **Samuel A. Nahulu**: Methodology; writing—reviewing & editing. **Emily L. Abrahamson**: Investigation; writing—reviewing & editing. **Mary P. Nivison**: Investigation; methodology; supervision; writing—reviewing & editing. **Kathryn E. Meier**: Conceptualization; data curation; formal analysis; funding acquisition; methodology; project administration; supervision; validation; visualization; writing—original draft preparation; writing—reviewing & editing.

## CONFLICT OF INTEREST STATEMENT

The authors have no conflicts of interest to disclose.

## ETHICS STATEMENT

There were no human or animal subjects utilized in this study. There is no material reproduced from other sources.

## Data Availability

The data that support the findings of this study are available on request from the corresponding author. The data are not publicly available due to privacy or ethical restrictions.
